# *“I wasn’t sure it would work. I was just trying”*: an ethnographic study on the choice of abortion methods among young women in Kilifi County, Kenya, and Atlantique Department, Benin

**DOI:** 10.1186/s12978-023-01720-x

**Published:** 2023-12-06

**Authors:** Gladys Akinyi Omondi, Jonna Both, Ramatou Ouedraogo, Grace Kimemia, Kenneth Juma

**Affiliations:** 1 African Population Health and Research Center, Manga Cl, P.O. Box 10787, Nairobi, Kenya; 2 Rutgers, Utrecht, The Netherlands; 3 International Rescue Committee, Nairobi, Kenya

**Keywords:** Abortion methods, Choices, Motivations, Providers, Abortion safety, Sub-Saharan Africa

## Abstract

**Background:**

Despite the increased availability of safe abortion methods in sub-Saharan Africa, women and girls continue to use unsafe abortion methods and procedures to terminate their unwanted pregnancies, resulting in severe complications, lifelong disabilities, and death. Barriers to safe abortion methods include restrictive laws, low awareness of safe abortion methods, poverty, and sociocultural and health system barriers. Nonetheless, there is a paucity of data on the decision-making around and use of abortion methods. This paper aims to provide answers to the following questions: Which abortion methods do women and girls use and why? Who and what influences their decisions? What can we learn from their decision-making process to enhance the uptake of safe abortion methods? We focus our in-depth analysis on the rationale behind the choice of abortion methods used by women and girls in Kilifi County in Kenya and Atlantique Department in Benin.

**Methods:**

We draw on data collected as part of an ethnographic study conducted between January and August 2021 on lived experiences, social determinants, and pathways to abortion. Data were collected using repeated in-depth interviews with 95 girls and women who had a recent abortion experience. Data from the interviews were supplemented using information from key informant interviews, focus group discussions, and participant observation. Data analysis was conducted through an inductive process.

**Results:**

Our findings reveal that women and girls use various methods to procure abortions, including herbs, high doses of pharmaceutical drugs, homemade concoctions, medical abortion drugs, and surgical abortion methods. Procedures may involve singular or multiple attempts, and sometimes, mixing several methods to achieve the goal of pregnancy termination. The use of various abortion methods is mainly driven by the pursuit of social safety (preservation of secrecy and social relationships, avoidance of shame and stigmatization) instead of medical safety (which implies technical safety and quality).

**Conclusion:**

Our findings reaffirm the need for comprehensive access to, and availability of, abortion-related information and services, especially safe abortion and post-abortion care services that emphasize both medical and social safety.

## Plain Language Summary

Despite the availability of safe abortion methods in sub-Saharan Africa, women and girls in the region continue to resort to unsafe methods, leading to severe complications, disabilities, and maternal death. This can be attributed to restrictive abortion laws, lack of awareness on safe abortion methods, poverty, and sociocultural and health system barriers. This paper uses data from a larger ethnographic study in Kilifi County, Kenya, and Atlantique Department, Benin, to understand which methods women and girls use, and why, to help improve the use of safe abortion methods.

Data were collected through in-depth interviews with 95 girls and women who had recently undergone an abortion, as well as key informant interviews, focus group discussions, and participant observation. The findings reveal that women and girls use various methods to terminate their pregnancies, including herbs, high doses of pharmaceutical drugs, homemade concoctions, medical abortion drugs, and surgical methods. They often use these methods once, multiple times, or in combination to achieve their goal. The main reason for their choice of methods is not medical safety but social safety, including preserving social relationships and avoiding shame and stigma.

We conclude that there is a pressing need for greater access to accurate, well-framed information about safe abortion methods. Abortion services should consider not only medical safety but also discretion to mitigate the social implications of having an abortion in a medical facility. By addressing these factors, it is possible to enhance the use of safe abortion methods and reduce the reliance on unsafe practices.

## Background

An estimated 25 million unsafe abortions take place every year, resulting in about 47,000 maternal deaths around the world [1]. Unsafe abortion is among the leading causes of preventable maternal death and illness within sub-Saharan Africa [2], which has the highest abortion case fatality rate of any region globally at roughly 185 deaths per 100,000 abortions, representing approximately 15,000 preventable deaths every year [3]. Additionally, unsafe abortion is responsible for a significant proportion of moderate to severe complications, such as incomplete abortion, sepsis or infection, hemorrhage, uterine perforation or laceration, damage to the genital tract, and multiple organ failure [4, 5]. Managing these abortion-related complications results in social and financial burdens for women and girls, communities, and health systems [6]. While restrictive abortion laws across sub-Saharan Africa are known to fundamentally drive the region’s high rate of unsafe abortions, other factors include religious and social stigma; limited awareness, agency, and autonomy; and health systems’ being unprepared to offer safe and quality abortion-related care [7].

Safe and comprehensive abortion care has been revolutionized in recent decades due to increasing health evidence, medical/health technologies, and human rights perspectives in sexual and reproductive health care [8, 9]. The efficacy and safety of simple technologies such as manual vacuum aspiration (MVA) and medical abortion drugs have made it possible for less specialized health professionals, such as nurses and midwives, to provide safe abortions [10]. Moreover, the use of medical abortion drugs has been proven to be safe and effective across diverse settings, whether self-administered or administered under the supervision of a healthcare provider [11]. The World Health Organization’s recent guidelines also recommend self-management approaches for medical abortion in whole or in part [12], which has expanded safe abortion choices for women, especially within restrictive contexts [13]. More recently, several sub-Saharan African countries have moved toward broadening legal grounds for abortion (i.e., when the physical or mental health of the mother is at risk and on socioeconomic grounds) to increase access to and the safety of abortions and also the quality of post-abortion care [14, 15]. Even so, abortion rates have remained relatively stable over the last 25 years [16]. Given the high rate of population growth in Africa and the increasing proportion of unintended pregnancies ending in abortion, the unchanging abortion rate implies an increasing number of induced abortions [3].

Several studies have shed light on the myriad factors continuing to drive unsafe abortion practices in different contexts within sub-Saharan Africa. In Ghana, for instance, Atakro et al. (2019) reported that limited knowledge of safe abortion services, poor socioeconomic conditions, cultural and religious beliefs, and stigma associated with unplanned pregnancies were key drivers of unsafe abortion [17]. Assessing how stigma affects abortion safety in Kenya, Rehnström Loi et al. (2018) revealed that social and gender dependencies influence women’s decision-making on abortion methods and often steer them away from safer abortion options. Further, women’s definition of “abortion safety” differs from the public health discourse, as women prioritize self-preservation, stigma management, reputation protection, and maintaining social relationships [18]. Consequently, they often avoid high-profile facilities that might increase their exposure to judgment and discrimination [19].

While these studies inform our general understanding of decision-making around abortion and the factors leading to the use of unsafe abortion methods, data are limited on the pathways toward using either safe or unsafe abortion methods, and especially the decision-making mechanisms around the choice of abortion methods. Analyzing how women and girls make decisions around the choice of abortion methods could be valuable in offering both practical and technical recommendations for improving the uptake of safe abortion methods.

### The Benin and Kenya contexts

While Benin does not have national data on unwanted pregnancies and abortion, the Guttmacher Institute estimates that 589,000 pregnancies occurred annually in Benin between 2015 and 2019, and of these, 227,000 were unintended and 84,300 ended in abortion [20]. Over the same period, the proportion of unintended pregnancies ending in abortion increased from 26 to 37% [21]. While the Ministry of Health estimates that 15% of maternal deaths result from unsafe abortions [22], few studies have described the context of unsafe abortion in Benin, signifying a critical research and knowledge gap that remains unaddressed [23]. Toward the end of 2021, the country reformed its abortion law to allow for abortion when the pregnancy is “likely to aggravate or cause a situation of material, educational, professional, or moral distress incompatible with the woman and/or the unborn child” [24]. Before that, including when we were conducting this study, abortion was only allowed when the pregnancy put the life or health of the mother at risk, as well as in cases of rape, incest, or fetus malformation.

By contrast, there is a substantial body of evidence on the challenge of abortion in Kenya [25]. The country’s 2010 constitution only allows abortion when there is a risk to the health or life of a woman or when there is a need for emergency treatment [26], although the High Court ruled in 2019 that abortion is allowed when the pregnancy is because of rape. Beyond these exceptions, the Kenyan penal code criminalizes abortion as a felony punishable by seven to fourteen years imprisonment for the client and the provider [27]. Social strictures exist as well. In Kenya, where many people identify as Christian, religious beliefs are both pervasive and influential in decision-making processes around abortion. Many religious groups oppose abortion, describing it as a sinful act [28].

This paper draws on data from an ethnographic study conducted in Kenya and Benin that aimed to explore the way women and girls navigate abortion decision-making in their specific social, cultural, and legal contexts. Certainly, there is value in comparing these two countries, which are both similar and dissimilar in areas such as abortion laws, health system structures, and access to and rates of self-managed abortions. In this paper, we specifically focus on describing the multiple methods of abortions used by women and girls, as well as the rationale guiding their choices. In doing so, we aim to better understand the complex factors driving women and girls toward unsafe versus safe abortions and how these factors intertwine to shape abortion pathways and outcomes.

## Methods

### Study design

The data presented in this paper were derived from a larger ethnographic study conducted between January and August 2021 in Kilifi County in Kenya and Atlantique Department in Benin. The broader study aimed to explore the lived experiences, social determinants, and pathways to (un)safe abortion of adolescent girls and young women in the two countries [29, 30]. This paper is a sub-analysis of the larger study that focuses specifically on describing the various methods of abortions that women and girls used, as well as the rationale guiding their choices.

### Study setting

The study was conducted in Kilifi County in the coastal region of Kenya and Atlantique Department in south-central Benin. Kilifi County is divided into seven sub-counties covering both urban and rural areas. For this study, data were collected in an urban area (Kilifi North) and a rural one (Kaloleni). By contrast, Atlantique Department is composed of eight communes spread across urban and rural areas. For this study, data were collected in a rural commune (Allada) and an urban one (Abomey Calavi). Both Kenya and Benin have restrictive abortion laws and policies [31]. Additionally, they have a disproportionate distribution of evidence around unsafe abortions and the availability of and access to safe abortion services, including post-abortion care [32].

### Study population and sampling strategy

We targeted 95 adolescent girls and young women for the study’s in-depth interviews (IDIs), 54 in Kenya and 41 in Benin; details on their sociodemographic characteristics are presented in Table 1.

**Table 1:** Sociodemographic characteristics of women and girls interviewed in IDIs

Characteristics	Frequency (n = 95)	
	Benin (n = 41)	Kenya (n = 54)
Age (years)		
15–17	8	8
18–24	21	41
25–30	5	5
31–40	7	0
Highest level of education		
No formal education	7	1
Primary school	7	18
High school	21	28
College	6	7
Area of residence		
Urban	10	12
Peri-urban	8	16
Rural	23	26
Marital status		
Married	8	3
Separated	5	4
Never married	28	47
Occupation		
Student	17	19
Employed/informal laborer*	22	19
Unemployed/housewife	2	12
Sex worker**	0	4

*Hairdressers, housegirls, bartenders, shopkeepers, waitresses, tailors, a casual worker at the cereals board plant

**Unique to participants in Kenya

We also conducted 69 key informant interviews (KIIs), 29 in Kenya and 40 in Benin (see Table 2). These key informants were stakeholders in sexual and reproductive health, including community health volunteers, community leaders, health providers (private/public), pharmacists and drug vendors, traditional birth attendants, and policymakers. The study also included 12 focus group discussions (FGDs) in Kenya: four with a mixed group of young men and women aged 18 to 24, four with mothers, and four with fathers aged 30 to 55 from the community. We conducted 15 FGDs in Benin: seven with a mixed group of young men and women aged 18 to 24, four with mothers, and four with fathers aged 25 to 65. While this manuscript mostly engages with material generated through the IDIs, data gathered through the other techniques provide a significant understanding of the social and cultural norms in which abortion experiences are shaped and help contextualize the IDI results.

**Table 2:** An overview of the key informants interviewed

Role	Kenya (n = 29)	Benin (n = 40)
Community health volunteer	4	4
Informal drug vendor	1	8
Pharmacist	5	4
Traditional birth attendant	3	9
Medical provider: public facility	4	4
Medical provider: private facility	2	3
Government official*	2	3

*County coordinator on reproductive, maternal, and childhealth, Ministry of Health representatives

The study applied a specific inclusion criterion for participant selection in order to recruit girls and young women of reproductive age who had a recent abortion experience, their male partners, the relatives of women or girls who had experienced an abortion, and the formal and informal healthcare providers from various facilities, informal drug vendors, pharmacists, and traditional birth attendants. To recruit the girls and young women with abortion experiences into the study, trained research assistants were stationed at specific primary health facilities across the two countries to identify girls and young women who had come for post-abortion care services. Research assistants also worked closely with community health volunteers and youth advocates for sexual and reproductive health and rights (SRHR) in identifying adolescent girls and young women who had experienced abortion in the past year. The community health volunteers and youth SRHR advocates were comprehensively briefed on the study and the ethical procedures at the initial stages of the study, and they supported the recruitment of girls and young women with recent abortion experiences. These were girls and women they interacted with in the process of their SRHR work; the volunteers and advocates sought approval to share the woman or girl’s contact information with the researcher and arrange a meeting. If the woman or girl agreed, she received an explanation of the study and informed consent, and then her consent was sought before inclusion in the study. The participating girls and women, in turn, occasionally connected the research assistants to their relatives (including fathers, mothers, sisters, grandparents, and aunts), provided these relatives were already aware of the abortion experience. IDIs with relatives and partners who were aware of the abortion experiences aimed to document their perspectives on the woman or girl’s abortion pathways, including the practices around pregnancy prevention, their reaction to the pregnancy, and their role in the decision-making process, as well as the care-seeking pathways. Informed consent was sought from these relatives before conducting interviews.

For the KIIs, especially with the health providers, participants were purposely selected based on their active role and involvement at the facilities in delivering post-abortion care/comprehensive abortion care services. For most of the other KII participants, including pharmacists and informal drug sellers, we employed the snowballing technique; we identified initial participants and they, in turn, proposed others who could be interviewed in the study. Regarding the FGDs, we identified participants with the help of community health volunteers and community elders.

### Data collection

Data were collected between January and August 2021 in both Kenya and Benin using repeated IDIs, FGDs, KIIs, and participant observation. For the IDIs, data-gathering entailed deep immersion in the service delivery processes at the facilities offering post-abortion care services to identify potential interview participants. Once prospective participants were identified and approached for purposes of informed consent, the research assistants created rapport with the girls and young women, and when possible, with their relatives and partners. IDIs were conducted using structured interview guides with a range of questions meant to explore intimate relationships, contraceptive (non)use, abortion decision-making, and abortion experiences.

The KIIs aimed to understand the role of key persons in abortion and post-abortion service provision in the county, including their rationale, challenges encountered, knowledge of policies and guidelines in abortion services provision, training, and equipment in health facilities, among other areas. The FGDs sought a broader perspective on adolescent reproductive health by including young people and the parents of adolescents, irrespective of whether they had experience with abortion. Specifically, the FGDs aimed to provide insight into the social and cultural dynamics in which abortion is embedded and covered topics like the transition to adulthood, perceptions of adolescent pregnancies and single motherhood in the community, parent-adolescent communication, reproductive decision-making, and induced abortion.

The IDIs, KIIs, and FGDs were conducted in various languages depending on the location. In Kenya, interviews were primarily conducted in Swahili, sometimes mixed with English. In Benin, the interviews were mainly conducted in Fon, Aïzo, and occasionally French. The number of follow-up interviews varied depending on the participant’s availability, the details of their lived experiences, and the value of following up for more information. The interviews took place in various settings that helped ensure privacy, such as health facilities or the participants’ homes, in private and quiet rooms, or outside.

### The research team and reflexivity

In both Kenya and Benin, we recruited a team of young female research assistants with backgrounds in anthropology and sociology to collect data and engage in the analysis and dissemination of the findings. Before data collection began, we took this group of young research assistants through a five-day, face-to-face training workshop that consisted of an introduction to the project, the study objectives and design, human research ethics (including the consent process and research on sensitive issues), and ethnography. The group of young researchers entered the field through an introduction by the head of targeted health facilities and community elders. Given the sensitivity of the study, it was introduced under a broader thematic approach (sexual and reproductive health) to protect the girls and women who chose to take part in the study and avoid stigmatization.

The research assistants were able to build rapport with patients and healthcare providers over an extended period of six months. They also took up other roles, such as assisting in entering records and helping patients to navigate the facility. To ensure objectivity, the research assistants discussed their progress and challenges during weekly debriefs conducted together with the wider research team (the principal investigator and research officers, who are also authors of this paper). The research assistants received a refresher workshop on the study objectives, ethics, and methodology halfway through their data collection, and they also had access to professional counseling sessions to deal with the stressful nature of events they witnessed, especially in the health facilities.

### Data analysis

The IDIs, KIIs, and FGDs were audio recorded with consent from participants, and the initial IDIs were transcribed and used as the basis for developing questions for follow-up interviews. Research assistants’ audio files and field notes were anonymized and then transferred to and stored in a Google Drive folder with two-step verification. Audio files were transcribed verbatim and translated into English and French. The research team coded the interviews following the development of a codebook that was based on the initial research proposal and themes emerging from the data. The coding process was done repeatedly to ensure newly identified themes were not missed in earlier rounds of coding. When a saturation point was reached and the data did not seem to provide any new codes, similar codes were gathered into the respective larger themes. For this paper, we focused predominantly on themes that described (1) the abortion methods used by women and girls; and (2) the motivations for using the specific abortion methods.

### Findings

**Fig. 1:**
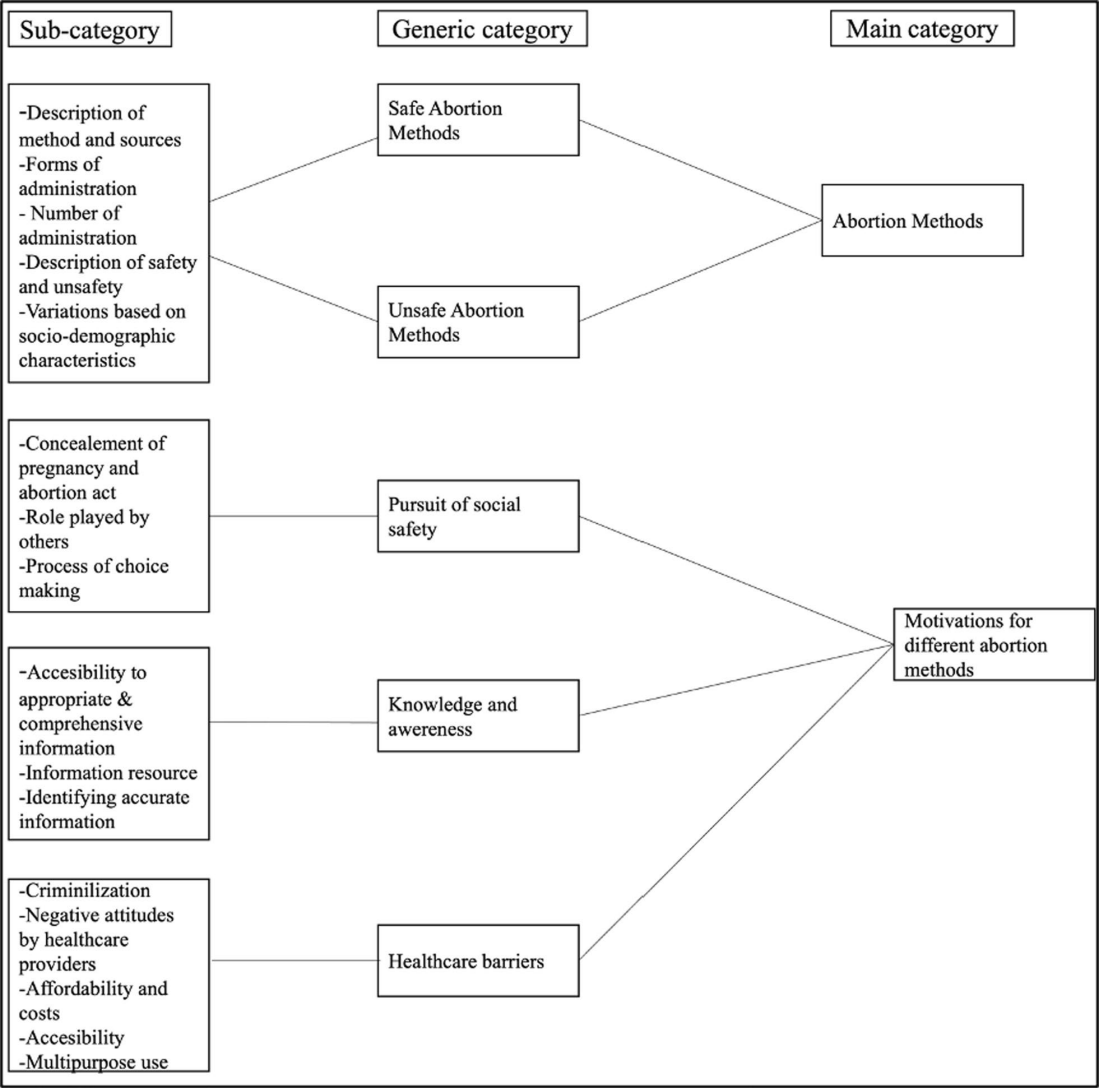
Coding tree describing the abstraction process

Two major themes emerged from our data: (1) the array of methods used by the participants to terminate pregnancies, and (2) the motivations for the specific methods that women and girls ultimately used to terminate pregnancies. We begin this section by presenting the methods used and then dive into the motivations, which include (a) the pursuit of social safety; (b) knowledge and awareness of different abortion methods; and (c) healthcare barriers. Figure 1 illustrates the abstraction process.

### Abortion methods

The analysis of participant interviews revealed that women and girls in Benin and Kenya used a variety of methods to terminate pregnancies, as summarized in Table 3.

**Table 3:** Summary of the abortion methods used by women and girls

Abortion method	Benin	Kenya
Herbs	Castor seeds, kodô, tisanes (herbal tea), hyssop leaves	Shubiri, mjaji, mkilifi, mwarubaini, aloe vera
Home remedies/ concoctions	Concentrated lime juice, sodabi, a mixture of Guinness and Moca, 7 Up, fresh coconut water, *la pottasse* (potash)	Boiled Coca-Cola, concentrated Quencher drink
Pharmaceutical/ unknown drugs	Sédaspir, Nivaquine, paracetamol	Unnamed hormonal drugs
Insertion of sharp objects	Wooden straw	–
Surgical abortion	Curettage, MVA	MVA
Medical abortion	Mifepristone, misoprostol	Mifepristone, misoprostol

As Table 3 shows, the methods ranged from homemade concoctions using local herbs to pharmaceutical drugs (drugs contraindicated in pregnancy or high doses of regular drugs, but not medical abortion pills) to medical abortion drugs and surgical abortion. In Kenya, women mentioned bitter herbs as being useful for terminating pregnancies (e.g., shubiri, mkilifi, and mjaji, which are succulent plants used to make herbal medicines), while in Benin, respondents cited means such as castor seeds, kodô (caïlcédrat tree bark or roots known to be bitter and used to treat different medical conditions), concentrated lime juice or sodabi (liquor made from distilled palm wine), hyssop leaves, and fresh coconut water. Apart from surgical abortion (MVA offered in healthcare settings or at medical providers’ homes, or curettage used by quack doctors) and sometimes medical abortion methods (administered in health facilities or at medical providers’ homes), all of the methods were administered by the women and girls themselves, usually through drinking them or inserting them into the vagina. In both Benin and Kenya, participants mentioned obtaining the herbs in their local surroundings, from street vendors, traditional healers, or through friends, relatives, or confidantes. For example:

*There was my friend who removed it* [pregnancy], *so when I was pregnant, I did not tell her I was the one, I asked what she used because there was someone who wanted to remove it. Then she told me she used leaves from the mkilifi tree. So, I went back home and boiled the leaves of mkilifi and drank it; then, I started bleeding.* (IDI, 17-year-old single girl, primary school student, urban Kilifi)

*I went to a lady who sells bush medicines and explained my case to her. She then sold me some. ...When I took the medicine there, I went to my little sister’s house, and I started to prepare and drink kodô.* (IDI, 32-year-old widowed woman, businesswoman, urban Benin)

Some participants said they used high doses of pharmaceutical drugs to terminate pregnancies, often over-the-counter drugs contraindicated in pregnancy. In Benin, for instance, participants commonly reported using Nivaquine (an over-the-counter antimalarial drug containing chloroquine) and Sédaspir (which is used to treat conditions such as apnea of prematurity, rheumatic arthritis, fever, and pain). Both drugs are ill-advised during pregnancy. Participants usually mixed the drugs with herbs or took high doses. In Kenya, some participants also reported using drugs meant to treat other reproductive issues, such as hormonal imbalance. As such, there was no guidance or recommendation on dosage, and the participants would take as much as possible until the drugs induced abortion. For example:

*I bought S*é*daspir first, and I went to the lady and I told her that I did not get my period today, and tomorrow it may not come. The lady told me to buy kodô and to go and prepare to drink it warm with the S*é*daspir tablets. …I said OK and I started, but the S*é*daspir that she sold me is not of good quality because it breaks into pieces by itself. I took a lot without any results. I understood that it was not effective because I did not get the expected results. I felt nothing in my belly, but there was still something moving. So, when I went to Houègbo, I looked for where they sell S*é*daspir there. A lady sold the three tablets at 500 [francs], and I bought kodô in addition, and I looked again for a leaf that looks red.* (IDI, 40-year-old divorced woman, businesswoman, urban Benin)

Homemade concoctions were common in both Benin and Kenya, especially among school-going adolescents, given that they are easily accessible and inexpensive. Some of the common concoctions that Kenyan participants reported included boiled soft drinks such as Coca-Cola and concentrated Quencher orange juice. In Benin, participants commonly indicated a mixture of alcoholic and soft drinks, such as Guinness and Moca, 7 Up, and *la potasse* (potash, a caustic substance) to terminate pregnancies:

*One of my friends told me I could drink concentrated juice since I didn’t have money. I went to the shop and bought the Quencher* [processed orange juice, usually diluted with water before consumption], *the small one. I went to my friend’s house and drank one glass. Immediately after drinking, I had a lot of pain; I was also bleeding a lot. It was very painful. I bled a lot for one week.* (IDI, 24-year-old single woman, college student, urban Kilifi)

*In our neighborhood, I had heard that a lady had an abortion, but they said she used 7 Up with something you call “akango”* [*la potasse*, or calcium chloride]*. ...And besides that, they said others use leaves and medicines that they prepare. That is why I tried the same.* (IDI, 19-year-old, single woman, student, urban Benin)

Some participants used medical abortion and surgical abortion as the first methods of pregnancy termination. During our interviews, participants who used medical abortion described the method using the route of administration (e.g., *“One drug is inserted in the vagina and another one under the tongue*”) but did not know the specific names of the drugs. A few participants reported having used surgical methods:

*I didn’t go far. It’s just here in the neighborhood, I went and told the doctor the situation I was in, and that I was begging for his help. Then he said now that I had decided, he would help me. So he put one drug in me, down there* [the vagina]*, and I then swallowed another one.* (IDI, 21-year-old single woman, waitress, peri-urban Kilifi)

*In the end, after the ultrasound, the doctor told me that the stage at which the thing* [pregnancy] *is, that it is better to do, I do not know* [what they call it,] *…aspiration or curettage or I do not know what.* (IDI, 23-year-old single woman, student, rural Benin)

As these excerpts show, participants chose medical and surgical abortion when they sought services at (private) health facilities and pharmacies and met with providers willing to help them, or they went to health workers they knew who offered abortion services from their homes.

Many women and girls used mixed methods to terminate a pregnancy or tried different ones (multiple methods) concurrently when they thought one method would not work effectively (see Table 4). Participants sometimes used the same abortion method multiple times or different methods sequentially or simultaneously. When they used multiple or mixed methods, herbs, pharmaceutical drugs, or homemade concoctions were often the first and most sought-after method. They would then reach out for medical or surgical methods by going to the hospital for post-abortion care once they experienced complications from their self-administered methods.

**Table 4:** Examples of the use of multiple and mixed methods

Age (years)	Country	Residence	Multiple/mixed methods used
22	Benin	Rural	Sédaspir + kodô
40	Benin	Peri-urban	First attempt: Sédaspir + alcohol, followed by Sédaspir + kodô Second attempt: kodô + Sédaspir Ibuprofen + kodô + Sédaspir herbs
23	Benin	Urban	First attempt: Two types of herbal plants + consultation with a traditional healer Second attempt: surgical abortion
23	Benin	Urban	Guinness + tisanes + paracetamol
19	Benin	Peri-urban	First attempt: salt water + Nivaquine + contraceptive pills Second attempt: surgical abortion
26	Benin	Peri-urban	Nivaquine + coffee
22	Benin	Rural	Nivaquine (five times) + lemon juice
24	Benin	Urban	First attempt: Cytotec Second attempt: Guinness Third attempt: unknown medicine
17	Kenya	Urban	Mixed methods: mjaji (herbal tree) + mkilifi
22	Kenya	Rural	Mixed methods: mjaji + mwarubaini (neem tree) + shubiri (herbal tree)
15	Kenya	Rural	Mixed methods: shubiri + mwarubaini (neem tree)
29	Kenya	Rural	Abortion pills (four attempts) + MVA
18	Kenya	Urban	One method multiple times: abortion pills (twice)
24	Kenya	Urban	Mixed methods: mix of traditional herbs + tea leaves
21	Kenya	Urban	First attempt: shubiri Second attempt: abortion pills Third attempt: MVA
22	Kenya	Urban	Multiple methods: aloe vera + shubiri + abortion pills
16	Kenya	Urban	Multiple methods: abortion pills + MVA

Women and girls in Benin and Kenya shared great similarities regarding their first choice of abortion method; in rural areas, herbs and homemade concoctions predominated, although in Kenya, medical abortion was also common in rural areas. Medical abortion pills were more commonly used in Kenya but were less typical in Benin. While more participants used high doses of pharmaceutical drugs like Sédaspir, Nivaquine, and paracetamol mixed with traditional herbs in Benin, our participants in Kenya rarely used high doses of pharmaceutical drugs. Additionally, the use of homemade concoctions differed: participants in Benin often mixed alcoholic and soft drinks to come up with drinkable potions, while those in Kenya reported drinking soft drinks either in concentrated form or after being boiled on high heat. While participants of various ages used homemade concoctions in Benin, such drinks were mostly used by school-going adolescents in Kenya. Only one participant, in Benin, used a sharp object.

### Motivations for different choices of abortion methods

#### The pursuit of social safety

Our participants opted for methods that would hide their abortion experiences from individuals in their community, such as friends, parents, and neighbors. According to a community health volunteer in Kenya, people refrain from seeking abortion services at health facilities because they fear being recognized by acquaintances. This notion is further supported by women and girls who expressed a preference for methods that disguise the act of abortion, often using herbal remedies that can be justified as treatments for various other conditions:

*If they go to a hospital, it will be known that so-and-so has gone to the hospital, to do what? …In most cases, the reason they go to those traditional healers is that it is a secret between him and the girl.* (KII, female community health volunteer, urban Kilifi)

*I used shubiri, I normally take it when I have stomach problems* [ulcers]*. I wasn’t sure it would work. I was just trying.* (IDI, 22-year-old single woman, college student, rural Kilifi)

In both countries, parents (especially mothers) and grandmothers were also seen as pursuing secrecy. Mothers were reported to help their daughters search for abortion methods to keep people (including their husbands and in-laws) from knowing about the abortion and avoid insults from community members:

*The mother is often afraid of insults from her husband or the family, so she quickly looks for the person who can help her without anyone knowing. She and her daughter go to the person. So, it’s only mum and daughter who know the secret.* (FGD, woman, parent of an adolescent girl, urban Benin)

As another example, one young woman in Kilifi explained how her grandmother provided mkilifi herbs to her:

*When I told my boyfriend, he said that pregnancy is not his; he did not make me pregnant. I have never been with another man. I told my grandmother that the man had denied the pregnancy. She thought it would be shameful if others heard about it. She then took mkilifi, boiled it, and gave it to me to drink. I drank and kept drinking many cups of water; then, I started bleeding.* (IDI, 21-year-old single woman, manual worker, urban Kilifi)

Male partners also sought social safety, which influenced their involvement in their partners’ selection of abortion methods. These male partners played diverse, significant roles in the process of procuring an abortion. Some actively chose the methods and accompanied their female partners to seek abortion services. However, the male partners’ decision-making on abortion method was often driven by their own desire to keep the pregnancy a secret, preserve their social standing, and in some cases, ensure the safety of their female partner and preserve their relationship. While some male partners guided their pregnant partner toward safe abortion methods, others took an opposing stance. The following excerpts illustrate these contrasts:

[My male partner] *took me to that old woman. It was scary. The woman said that I have to accept to either die or live because there are different pregnancies: some are difficult, and others are easy.* (IDI, 21-year-old single woman, household help, urban Kilifi)

*When I told my partner that I was pregnant, he was shocked, so I asked him what we were going to do. He then told me “Then let us remove it.” He then looked for a doctor for me. But then, the pregnancy was. The doctor told me if I insert these drugs, it won’t come out because it was already big. I had reached three months. He had to use the other method, that of “kuvuta mtoto ile na ile chuma”* [pulling the baby, that one with the metal]*. I felt a lot of pain, I also lost a lot of blood. That thing is very risky.* (IDI, 18-year-old single woman, student, peri-urban Kilifi)

*Knowledge and awareness of different abortion methods* Sometimes, the choice of an abortion method depended on the woman or girl’s knowledge. Before contemplating an abortion, most women and girls did not have sufficient information on the existing abortion methods; they often had heard of specific herbs that would work or of “abortion pills,” which are sometimes confused with emergency contraceptive pills. Abortion is highly taboo among these communities, and the accessibility of appropriate, comprehensive information on abortion methods remains a challenge:

*Before my own experience, I had not heard about how people end pregnancy nor knew of anyone who had done it.* (IDI, 18-year-old single woman, apprentice, rural Atlantique)

*It was one month. Because I started feeling unwell and wondered what was wrong with me. That’s when I tested and saw two lines. Then, I started asking around what people do when they get into such situations, and people told me different things. After, they told me that I should start with the shubiri*. (IDI, 24-year-old single woman, bar waitress, rural Kilifi)

Before pregnancy, girls and women were often unaware of ways to terminate the pregnancy, and the information they did have was vague and based on hearsay. In Benin and Kenya, the only thing many young women knew was that one could die from an abortion, as they witnessed this in their community or heard about it from their mothers. In addition, there is little knowledge about safe abortion methods like medical and surgical abortion:

*I did not think of going to the hospital because I hadn’t sat down with anyone to give me a hint of how they abort pregnancies at the hospital. I didn’t know what they used, drugs or something else. So, when I heard that shubiri can work I thought to try it because I hadn’t heard anyone else’s experience of how they do it at the hospital.* (IDI, 21-year-old single woman, student, urban Kilifi)

*What I knew about abortion is what I learned in the pharmaceutical field. I didn’t know if there are traditional things that are used for abortion. I just knew that there are abortion drugs. But I was afraid to take them because they can cause death.* (IDI, 26-year-old woman, seller of drugs at a pharmacy, urban Benin)

In seeking information and methods to terminate their pregnancy, women and girls rarely sought multiple sources of information before deciding which method to use. The secrecy surrounding unwanted pregnancy and the stigma around abortion prevent a thorough search for information and careful evaluation of options. Women and girls would rather go for the advice they can obtain from that one person they trust enough to ask. In such situations, women and girls ideally try to confide in someone they know has procured an abortion herself because she won’t judge them and will advise them on a “safe,” effective abortion method:

*I believed the girl because it’s something that she experienced. ...Also, she was someone close, and it’s as if she’s also a relative, so I said, “She can’t want this thing to harm me, so if it works, it works; if it won’t, then okay.” I was ready for anything.* (IDI, 22-year-old single woman, student, rural Kilifi)

Our participants hardly mentioned the internet and helplines as useful resources for information on abortion. For example, one participant in Kenya tried to use a helpline but said no one picked up the call despite numerous attempts. Only two participants in Benin mentioned having obtained information on abortion methods online: one said her partner had described the surgical abortion method to her as painful and she needed more information on the procedure, while in the second case, the partner used the internet to find information on general topics but ultimately relied mostly on his friends within the healthcare profession for guidance:

*The internet told me when it’s in the beginning, I think, up to two months, some medications make it go away. …The internet informs me a lot, but now, I have a lot of friends in medicine.* [child cries] *I have a lot, a lot, a lot, a lot, so when I have a little situation, I call them: “Hello! I have such-and-such a problem. How is it managed?”* (IDI, 36-year-old male partner, technical officer, rural Benin)

*I went on the internet, looked up the different abortion methods that exist, um, drugs that could induce abortion, which ones you could get without a prescription, and I found in my research that everything that could do the job was necessarily under prescription*. (IDI, 28-year-old married woman, facilitator, urban Benin)

In both excerpts, educated young people found trustworthy information about safe methods (suction and medical abortion). However, the internet may also refer users to unsafe methods, such as herbs and concoctions, as these are widely promoted online (on Benin’s Facebook pages, for example).

Ultimately, most women and girls end up depending on their peers, female relatives, vendors, and friends for information and advice on medication, and they are often referred to traditional methods, pharmaceuticals not intended for abortion, and/or homemade concoctions. These trusted individuals might also know someone who can help in acquiring abortion methods, including pills and even surgical methods. Notably, however, information from friends and relatives is highly diverse, fragmented, and based on other women and girls’ own experiences and knowledge, making it highly heterogeneous and different from medical standards.

*Healthcare barriers* Interviews revealed how abortion is perceived as a criminal act, and girls and women known to have procured an abortion face the risk of arrest and prosecution. When some women and girls presented at health facilities with abortion-related complications, health providers threatened to call the police. Additionally, women and girls faced harsh judgment and maltreatment from healthcare providers when they presented any abortion-related issues:

*Yes. I had gone to pay. I went downstairs to pay. When I was down there paying, they saw what the paper explained, and they said, “You schoolchildren come here after abortion and come here for blood transfusion. Wait here; we call the police.” When they were about to call, there was another mother who said, “Don’t do that. She is paying the money.”* (IDI, mother of an 18-year-old primary school student, rural Kilifi)

*If I go to a hospital, I will be told I am a murderer; they will tell me a lot of bad things.* (IDI, 20-year-old single woman, manual worker, urban Benin)

However, legal restrictions on abortion do not only apply to women and girls. Healthcare providers in some cases decline to offer pregnancy termination services for fear of prosecution. Religious and personal beliefs also influence healthcare workers’ attitudes toward abortion patients. Some healthcare providers displayed negative attitudes toward abortion patients and declined to offer abortion services such as MVA, saying it is a "dirty job" and that their religious beliefs do not allow them to perform abortion procedures. Participants also said some healthcare providers (especially in Kenya) discriminate against patients seeking abortion services (e.g., uterine evacuation using MVA after an unsuccessful abortion attempt) and prioritized patients seeking other health services. Interviews and observations with providers also indicated that most of the time, these providers conduct painful procedures such as MVA with no effort to manage patients’ pain. KII interviewees noted:

*I do not offer…abortion services because my faith does not allow it.* (KII, male health care provider, public facility, urban Kilifi)

*That thing* [MVA] *is very painful. We do it as if it is not painful, but it is very painful. I don’t like those dirty jobs. …The blood that comes out of it is dirty; you know, as doctors, we like clean blood, fresh blood. But those come out as clots. I don’t like that.* (KII, female healthcare provider, public facility, urban Kilifi)

Moreover, women and girls—especially in Kenya—associated health facilities with unsafe abortion methods based on stories they had heard of other women who died getting an abortion from a health facility. This could be explained by healthcare providers having insufficient training to perform an abortion in the past. Despite changes and reforms in the medical sector, women and girls remain unaware of safe abortion methods offered in health facilities.

Affordability and healthcare costs are also crucial determinants of women and girls’ choice of abortion method in Benin and Kenya. Due to the legal restrictions in both counties, most abortion services among respondents were clandestine. Therefore, the prices could not be regulated and were often raised by abortion providers and clinics depending on how desperate those seeking abortion services were. The costs of medical and surgical abortion in both Benin and Kenya were relatively high, as described by our participants. In Benin, most participants reported paying between 25,000 and 100,000 francs (US$40 to $160) to get either a medical or surgical abortion, while in Kenya, participants needed between 3000 and 20,000 Kenyan shillings (US$25 to $160). Herbs were comparatively cheap, 300 to 1000 francs (US$0.50 to $1.60) in Benin and 50 to 100 Kenyan shillings (US$0.40 to $0.80) in Kenya, or participants would find these herbs for free around their homes. Most of the women and girls in our study reported that they resorted to homemade concoctions and traditional herbs because they were cheaper than medical and surgical abortion. Additionally, women and girls often depended on their partners to cover the costs of abortion, meaning their choice was limited by the amount of money received from their partners:

*If I had my own money, I would have gone to the hospital.* (IDI, 21-year-old single woman, household help, urban Kilifi)

*The lady said that she is going to take me to a guy but I am going to have to find 2,000* [francs] *first, and I said that I don’t have any money, and if it is so, that I have 1,000* [francs] *like that, and I handed that to the lady. But when I gave it to the lady, she didn’t take me to the guy anymore; she made me the tea, and I drank.* (IDI, 18-year-old single woman, student, rural Benin)

Further, there was substantial misinformation and discrepancies on the standard costs of medical and surgical abortion methods. Our participants reported having been deceived by healthcare providers and social actors within their network to pay more money than they should for an abortion. On other occasions, some girls took too long to raise money to successfully visit a health facility and ended up using unsafe abortion methods that were cheaper, a practice that may lead to unsuccessful and repeat attempts:

*The first doctor, I used 3,000 [shillings]; the second, I used 1,500; and the third one, I used 3,000, later add[ing] 1,500 shillings… So in total, I used 9,000 shillings. I just felt bad when I remembered the cost I had incurred without success.* (IDI, 29-year-old single woman, unemployed, rural Kilifi)

## Discussion

In this article, we set out to understand why women and girls are continuing to use unsafe abortion methods despite the increased availability of safer abortion methods in sub-Saharan Africa. We looked at which abortion methods that women and girls used in Kenya and Benin and studied their underlying rationale, including who and what influenced their decisions, to learn from women and girls’ decision-making process and enhance the use of safe abortion methods.

Our findings present a variety of methods used by women and girls in Kilifi County, Kenya, and Atlantique Department, Benin. These methods include homemade concoctions using local herbs, high doses of pharmaceutical drugs (not abortion drugs), medical abortion drugs, and surgical abortion. Apart from medical abortion drugs and surgical abortion, the methods used differed between Kenya and Benin. For example, in Kenya, women and girls mentioned using bitter herbs and homemade concoctions, including boiled soft drinks. Notably, homemade concoctions were more prevalent among school-going adolescent girls in Kenya due to their accessibility and low cost. By contrast, women and girls in Benin mentioned using local herbs, seeds, and roots; concentrated juice; or strong liquor. Pharmaceutical drugs that are contraindicated in pregnancy were also common and were often mixed with herbs or taken in high doses.

Notably, in both countries, unsafe abortion methods were more common than safer ones. This finding is in line with other recent studies showing the high prevalence of unsafe abortion in sub-Saharan Africa despite the increased availability of safer abortion methods [10, 33]. Contrary to other studies [34, 35] reporting that women commonly use sharp objects to procure an abortion by inflicting direct injury to the vagina or inserting foreign objects (e.g., coat hangers, metal, bones, or twigs), only one woman in our study, in Benin, reported using sharp objects to procure an abortion. The Kenya field researchers attributed this to the wide availability of abortive herbs growing in Kilifi; when carrying out a different research project, they had identified the use of sharp objects as more common in some other regions of Kenya. However, the herbs, high dosage of pharmaceutical drugs, and homemade concoctions that most women and girls in our study used are just as dangerous as invasive techniques [36]. In addition to the potential for incomplete abortion that risks women’s lives, these methods’ effects and potential harm have not yet been fully established, a concern that has been expressed by other studies examining the use of herbs for abortive intent [37, 38].

Additionally, it is worth noting that in both Kenya and Benin, the choice of abortion methods was not based on any scientific knowledge or evidence. Participants often relied on limited information or hearsay from friends, family members, or community members. The lack of comprehensive information about safe abortion methods, such as medical or surgical abortion, contributed to the use of potentially harmful methods. A recent review on the next “infodemic” expresses the devastating effects of abortion misinformation on maternal mortality and larger-scale policy on abortion [39]. Several other studies have also cited a lack of information as a key barrier to safe abortion [40, 41]. Our participants rarely used the internet to search for information or used hotlines that help with abortion dilemmas. In places that have hotlines, these are sometimes not fully functional and, hence, unreliable; at other times, they face limitations, such as language barriers [42].

Accordingly, the pursuit of social safety was a key motivation for women and girls’ choice of abortion method in both Kenya and Benin. This meshes with Kebede et al.’s (2019) study on negotiating the social and medical dangers of abortion in Addis Ababa, which emphasizes women’s strong motivation to preserve their social safety rather than their physical health [43]. This issue is also considered paramount by other social actors in women and girls’ lives who play a key role in their abortion decision-making. These actors provide financial support, information on different methods, and social support, all intending to preserve social safety. Mothers, for instance, want to maintain their social standing in society and avoid shame by choosing methods that conceal their daughters’ abortion experiences. Partners are often motivated by the same concerns. These motivations can be explained by the stigma and discrimination surrounding abortion. Other studies have also reiterated how stigma shapes women’s abortion experiences [44–46].

Healthcare providers’ attitudes and beliefs also had a significant influence on participating women and girls’ choice of abortion method. Our findings show the lack of willingness of some medical providers to provide abortion services. These echo the findings of a systematic review by Rehnström Loi et al. (2018) on healthcare providers’ perceptions and attitudes on induced abortions in sub-Saharan Africa and Southeast Asia, which suggest that some medical providers’ unwillingness to provide comprehensive abortion services is associated with the stigma and discrimination surrounding abortion [47]. This means providers offering abortion suffer stigma and discrimination in and outside their workplace [48]. However, their lack of willingness and beliefs that conflict with national law drive women away from seeking abortion in the hospital [49].

## Conclusion

Our study highlights the variety of methods used by women and girls in Kilifi County, Kenya, and Atlantique Department, Benin. Importantly, it also describes these women and girls’ motivations to choose specific abortion methods. While different factors come together to shape women and girls’ choices (like their knowledge of different abortion methods, affordability, and availability), the pursuit of social safety is paramount. Importantly, the pursuit of social safety matters not only to the woman or girl but also to the social actors surrounding her who are key in influencing the choice of method. Therefore, interventions geared toward ensuring safer abortion should pay more attention to ensuring women and girls’ social safety. There should also be a variety of channels employed to share reliable information and knowledge on the availability and efficacy of safe abortion methods. It is particularly important to make sure information reaches women and girls in rural areas, where there are often high levels of illiteracy and challenges in accessing the internet. However, information dissemination should not be limited to women and girls alone but also include other actors, such as partners, parents, and friends, who are key references for women and girls and influence their decisions.

## Data Availability

The data generated from this study are not publicly available because of the large amount of direct and indirect identifiers that the data set contains and the sensitivity of the subject in both countries in which the study was conducted. To access anonymized data generated in this study, a request can be made to the authors. Availability will be subject to signing a data confidentiality agreement.

## Abbreviations

FGDs: Focus group discussions
IDIs: In-depth interviews
KIIs: Key informant interviews
MVA: Manual vacuum aspiration
SRHR: Sexual and reproductive health and rights
